# Living with presbyopia: experiences from a virtual roundtable dialogue among impacted individuals and healthcare professionals

**DOI:** 10.1186/s12886-022-02432-9

**Published:** 2022-05-05

**Authors:** Mile Brujic, Paola Kruger, Jeff Todd, Elizabeth Barnes, Mark Wuttke, Flavia Perna, Jorge Aliò

**Affiliations:** 1 Premier Vision Group, Bowling Green, OH USA; 2 EUPATI, Rome, Italy; 3 Prevent Blindness, Chicago, IL USA; 4 Novartis AG, Basel, Switzerland; 5 Universidad Miguel Hernández and Vissum Miranza, C/ Cabañal, 1, 03016 Alicante, Spain

**Keywords:** Patient life experience, Unmet need, Mode of action, Clinical trials, Interactive dialogue

## Abstract

**Background:**

Presbyopia is a common progressive vision disorder characterised by an inability to focus on near objects. The emergence of newer treatment options in addition to spectacles or contact lenses highlights the importance of assessing patient/user preferences.

**Methods:**

People with presbyopia and healthcare professionals (HCPs) took part in a moderated, structured discussion of specific questions on a virtual advisory-board platform. The objective was to better understand unmet needs and the experience of living with the condition. Closed and open questions were included.

**Results:**

Nine individuals (age 40 to 70 years) with presbyopia participated, from Australia, China, France, Italy, Ireland, Japan and the US. One ophthalmologist and one optometrist represented the perspective of HCPs. Over two weeks, 621 posts were entered on the platform. There was widespread agreement that the often stated association between age and presbyopia was unfortunate. Some participants had developed presbyopia at 30–45 years of age. What is more, the association with age was seen as implying a natural process, reducing the incentive to treat. Instead there was a call for an action-oriented view of presbyopia as a condition which may be effectively treated in the future. All participants experienced dealing with presbyopia as burdensome, affecting quality of life to varying degrees. When considering new treatments, convenience was the most important factor. The option to administer drops when needed was considered favourable, but short-acting treatments may not reduce inconvenience compared with spectacles. Participants viewed a therapy that targets the underlying cause of the condition favourably compared with symptomatic treatment. Side effects would severely reduce the appeal of drops. For clinical trials in presbyopia, patient-reported outcomes should be mandatory and need adequately to capture quality of life. Studies in presbyopia must be designed to minimise the inconvenience to participants in order to counter the risk of high drop-out rates.

**Conclusions:**

The interactive format provided insights into living with presbyopia, particularly the negative impact on quality of life, subjects’ openness to new therapies, and the need to move away from considering the condition an unavoidable and intractable consequence of ageing.

## Plain English summary

The term presbyopia describes the difficulty to focus the eyes on things nearby, due to stiffening of the eye lens. The condition often considered something which worsens with increasing age. Many people cope with presbyopia by wearing reading glasses or bifocals, but alternative treatments are being developed. This publication reports from a moderated discussion among people with presbyopia and healthcare professionals specialising in eye health. People with presbyopia strongly felt that it should not be seen as an inevitable effect of middle age, but as something which may be treated medically. They felt that reading glasses, bifocals and monovision lenses were a daily burden and did not fit with how they wanted to live their lives. When discussing possible medical treatments, the option to use drops instead of glasses to improve eye sight appealed to the participants, particularly if the drops acted on the mechanism behind the stiffened lens with effect over many weeks or months. Convenience was the key benefit the participants would look for when considering a new treatment. Importantly, drops must not have any undesirable effects such as burning. The roundtable discussion showed the need for both healthcare professionals and those living with presbyopia to take the condition seriously with an an action-oriented view towards better therapies in the future.

## Introduction

Presbyopia is a progressive vision disorder characterised by an inability to focus on near objects. The primary cause is likely to be an increase in lens rigidity [1, 2], although factors such as ultraviolet radiation have been suggested to contribute to premature ageing of the lens [3–5]. Presbyopia is very common: it has been estimated that more than four persons out of five aged > 40 years will develop presbyopia and that by 2030, more than 2 billion people globally will suffer from the condition [6].

The most common way to correct presbyopia is with spectacles or contact lenses, both fixed- and variable focus systems. However, not only uncorrected presbyopia, but also the dependence on corrective visual aids such as reading glasses have a significant impact on quality of life [6–9]. Moreover, wearers of multifocal glasses have a high risk of falls because of apparent displacement of fixed objects at different parts of the visual field [10]. There is thus an unmet need for alternatives, including more convenient treatments. Currently, surgical options include laser surgery, refractive lens exchange or micro-insert scleral implants [11, 12]. Non-invasive, locally applied agents to improve accommodation in humans are the focus of increasing research attention. These include miotics which mediate contraction of the pupil to provide symptomatic relief [13, 14] and lipoic acid choline esters which act on disulphide bonds between crystalline lens proteins, thereby targeting the underlying cause of presbyopia to potentially soften the lens [15].

As more treatment options emerge, the question of patient/user preferences becomes important [16]. The opinion of patients may be very different from those of their physicians, as has been noted for a number of illnesses [17]. For people with presbyopia, little is known about attitudes, partly because of a historical lack of focus on the condition among clinicians and healthcare researchers, and partly due to a widespread view of presbyopia as a condition which can be ameliorated satisfactorily by simple, often low-cost, reading glasses.

A number of methods are employed for assessing patient experiences, often using qualitative interviews and quantitative questionnaires [18, 19]. A recent addition to the toolkit is social media listening, which has been applied successfully in a range of eye conditions including presbyopia to identify topics patients consider important in peer-to-peer discussions [9, 20, 21]. A limitation of such research is that it may over-represent the patient side in isolation, while missing insights into how views can be exchanged and modified in dialogue with HCPs.

To better capture the dialogue element, we used a virtual advisory board platform to conduct an interactive, moderated, structured discussion of specific questions between people with presbyopia and HCPs. The objective was to better understand the life experience of people living with presbyopia, unmet needs and views on emerging non-surgical treatment options, as well as thoughts on the design of clinical trials of novel medical presbyopia treatments from potential participants’ viewpoint.

## Methods

### Participants and discussion platform

The structured discussion took place in June 2021 on a virtual advisory board platform (Within3, Lakewook, OH, USA). Nine representatives with presbyopia and two HCPs were included. Participants viewed resources (guiding questions; patient materials from the National Insitite of Health; general background presentations on presbyopia by HCPs; examples of emerging alternative treatments including miotics and lipoic acid choline esters; schematic clinical trial designs) within the platform, which they could access from any connected device at any time that suited individual schedules. All responses and comments were visible to participants who could provide input at all stages of the discussions. The session moderator was an independent communications professional and did not take part in the discussion. The moderator had access to patient responses and could provide clarification or ask for additional information where appropriate.

Participants were recruited through their HCPs and from eye-health focused patient advocacy groups, with the goal of including a mix of age groups (targeted range 45–70 years), sex and ethnicity. The targeted participant profile was pre-specified as currently living with presbyopia requiring the use of reading glasses, prescription glasses or contact lenses; a history of these aids for the presbyopia for between 2 and 10 years; no other concomitant eye condition or disease except for refractive conditions and/or Dry Eye Disease. All participants were informed on the objectives of the project and future use of the results, and provided written, informed consent to take part in the roundtable discussion. The HCPs were identified based on internationally recognised expertise in the field of presbyopia and a record of peer-reviewed publications on the subject, with the aim of participating HCPs representing the perspectives of ophthalmologists as well as optometrists. HCPs took active part in the discussion and answered questions from the panellists, but they did not participate in answering the closed and open questions which structured the discussion.

### Discussion topics and analysis

Discussions focused on three topics: life experience of people living with presbyopia; views on emerging medical treatments offering symptomatic relief or targeting the underlying cause of the condition; and views on the design of clinical trials of medical presbyopia therapies from a participant perspective.

A combination of closed and open questions were included. Examples of the former are ‘*Which health care professional did you first consult about your presbyopia?*’ or ‘*How burdensome do you feel your presbyopia symptoms are?*’ Open questions concerned matters such as ‘*How do you feel presbyopia is best described?’ or ‘What do you believe are the most important patient needs in the management of presbyopia?’.*

All data were analysed descriptively. As this was a qualitative study, there was no a priori hypothesis. Closed question results are presented numerically. Representative quotes from the participants are in italics.

## Results

### Participant characteristics

Nine individuals with presbyopia, from Australia, China, France, Italy, Ireland, Japan and the US, took part in the virtual roundtable discussion. Three participants were also active in patient advocacy in eye-related and other disease areas. Two individuals had experience of taking part in clinical trials, for conditions other than presbyopia. The age of the participants ranged from 40 to 70 years; the experience of living with presbyopia from 3 to 20 years. One ophthalmologist (JA) and one optometrist (MB) represented the HCP perspective. Over the roundtable period a total of 621 posts were entered on the platform.

### Participant life experience

Overall, participants’ initial contact with HCPs focused on optometrists, ophthalmologists and opticians, with optometrists the most common profession. General practitioners or family doctors were not among those consulted. After the initial diagnosis, regular HCP visits were typically felt unnecessary unless symptoms worsened.

**Fig. 1:**
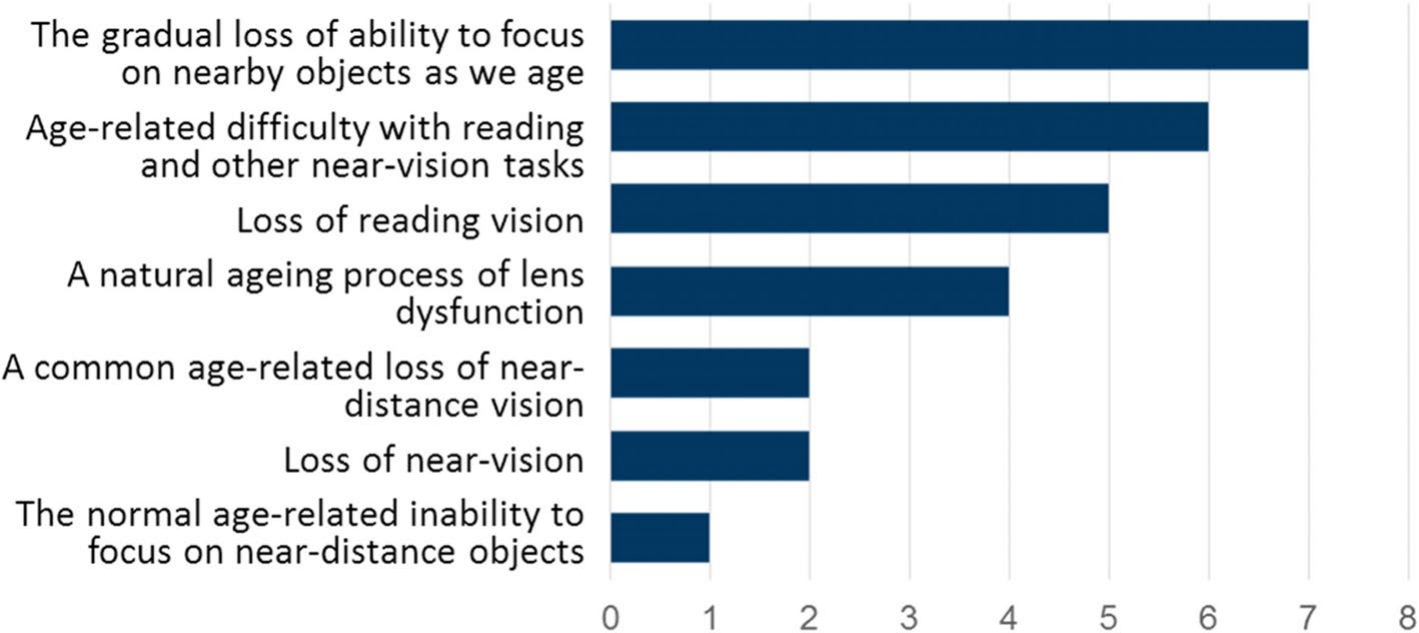
Preferred definitions of presbyopia (3 mentions per respondent possible)

When asked for their preferred definition of presbyopia, terms associated with age were frequently chosen (Fig. 1). However, there was widespread agreement among the panel that the stated association between age and presbyopia was unfortunate, for several reasons. First, some participants had developed presbyopia at 30–45 years of age. Secondly, in the opinion of the panel the association with age implies a natural process, reducing the incentive to treat. To avoid a sense of futility, participants preferred to view presbyopia as a progressive, treatable disease in its own right:

‘*Presbyopia is not just a to-be-expected result of middle age, but an eye health condition that can have multiple interventions’*

In initial discussions with HCPs, the term ‘presbyopia’ was rarely used in the panel’s experiences. Overall, it was felt that additional insights or options were insufficiently provided by HCPs. Downplaying of the seriousness of presbyopia was indicated as a possible barrier to an informed dialogue between patients and HCPs about the condition and therapeutic options.

All participants experienced dealing with presbyopia as a burden, but the severity varied between individuals. Current treatment options, mostly commonly prescription and over-the-counter reading glasses, were seen as acceptable, but few participants were fully satisfied. Even with reading glasses, there were several reports of eyes tiring easily during reading.

The panel shared resentment of becoming dependent on their visual aids. The need to juggle a number of visual aids in the course of a typical day was considered a major inconvenience, in particular by those who needed more than one pair of spectacles for different distances and activities. Frequently forgetting and having to search for glasses was a common frustrating experience. Several members needed additional glasses or contact lenses for astigmatism or other conditions, which compounded the inconvenience. An additional burden was the need for eye examinations and to adapt to new, adjusted strengths of glasses every few years.

### Views on emerging treatments

The panel welcomed the scenario of future therapies, as ‘*the options of reading glasses, bifocals and monovision lenses don’t fit how I want to live my life.*’ The discussion centred on two emerging treatments, short-acting miotic drops and longer-acting crystalline lens targeting lipoic acid choline esters.

The most important factor when considering new treatment was convenience, reflected in the statement that they ‘*may provide various options to improve quality of life’*. A miotic was thought to be suitable for occasional use in conjunction with glasses or contact lenses rather than as daily replacement for visual aids. The option to administer drops when needed was noted as a positive. (‘*No more fussing. Just two drops and let’s go!* ‘) A drawback was that short-acting treatments may present the user with the same inconvenience (e.g., multiple vials) as spectacles. A possible initial treatment regimen over days or weeks followed by long-term (weeks or months) relief from the need to wear glasses may be more convenient. Targeting the underlying cause of the condition was considered an appealing concept compared with symptomatic treatment. But the panel noted that the two approaches were not mutually exclusive. Importantly, side effects would severely reduce the appeal of drops.

**Fig. 2:**
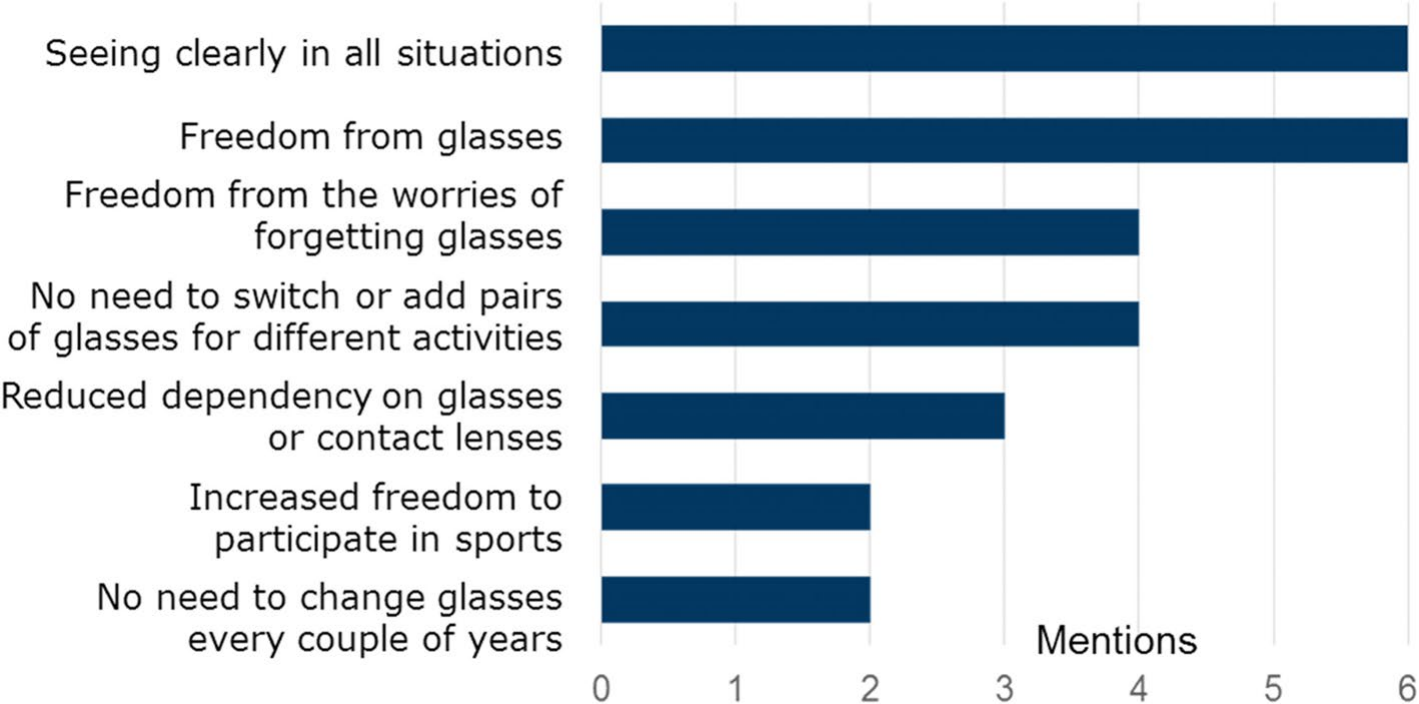
Key potential benefits of eye drops in the view of people with presbyopia. (Multiple mentions possible)

The greatest potential benefits of eye drops were freedom and seeing clearly in all situations without glasses (Fig. 2). The participants considered people with presbyopia unlikely to add one more process to their regimen without experiencing large benefits. By the same token, those who need glasses for other conditions in addition to presbyopia would benefit less from drops. There was consensus that the greatest interest in an emerging treatment would come from people with newly recognised presbyopia who have difficulties reading but have not been using visual aids for any extended period.

### Design of clinical trials of medical presbyopia therapies

‘*I think we who are affected by this condition should not settle for coping with it as best we can, but we should encourage the development of alternative treatments.*’

The panel provided insights into the design of presbyopia trials with topical therapies from the perspective of potential study participants. In line with the thoughts on who would derive the greatest benefits from drops, people with newly recognised presbyopia but without a history of wearing glasses were considered most motivated to take part in a trial. Excluding subjects with dry eye disease may be appropriate to reduce confounding effects as these subjects have a high use of drops for their condition. But the panel were concerned that since dry eye disease disproportionately affects women [22] this might reduce the ability to recruit female participants.

Beyond improved vision or accommodation, end points should focus on the patient experience. Patient-reported outcomes (PROs) need adequately to capture quality of life. Table 1 shows suggested specific measurements to be kept in mind when evaluating existing instruments and those in development.

‘*Vision tests are fine for assessment but the bottom line is quality of life*’

**Table 1:** Suggested PRO measurements for trials of medical presbyopia therapies

How many times have you been unable to do each of the following over the last 24/48 h	Read a book/newspaper
	Read medicines instructions
	Read menus, ingredients
	Distinguish between shampoo and conditioner in shower
Were you prevented from doing something because of glasses/lenses?	Rubbing eyes
	Cleaning make-up
	Opening eyes under water
Actual physical effects of drops	Stinging
Patient acceptability	Cultural
	Religious
	Ethical (animal testing)

**Fig. 3:**
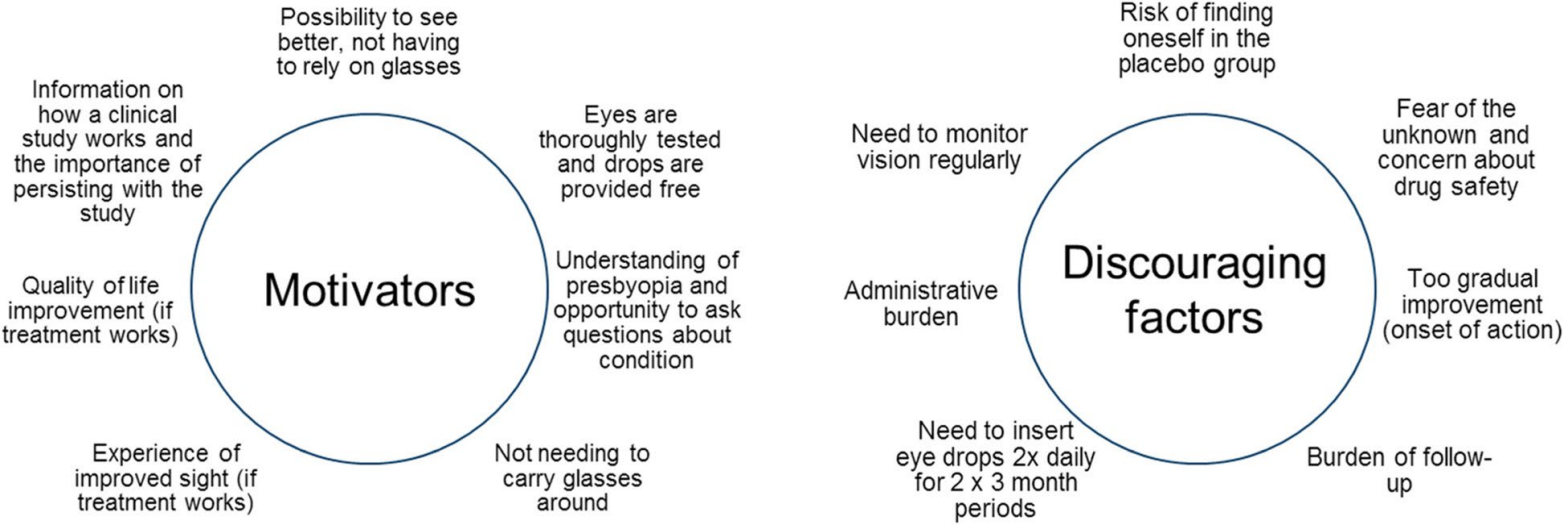
Motivating and discouraging factors for people participarting in a clinical trial of medical presbyopia treatments

The panel identified a number of motivating and discouraging factors for trial participants (Fig. 3). Specific suggested support to minimise discontinuations is listed in Table 2. The additional inconvenience of taking part should be minimised, e.g., by a limited need for eye tests to around 3 min 1–2 times/week. The panel welcomed the suggestion of an app to enable eyesight self-testing as a simplified and self-empowering tool. Information, such as shared experiences from participants in other clinical trials, was highlighted as an aid to retain study participants. Such information should include feedback on sight and test results during the trial, rapid and easy reporting of side effects, and continuing safety updates.

**Table 2:** Suggested support to minimise discontinuation in a trial of medical presbyopia treatments

Support to counter ‘trial fatigue’
▪ Psychological support
▪ Engagement as a true science participant partner and not a ‘study subject'
▪ Support with day-to-day organisation on visit days
▪ Option to ask questions of study investigator/ study nurse
▪ Free or discounted routine check-ups
▪ Simple automatic reminders to keep people on track
▪ Minimal number of interventions and tests to minimise disruption
▪ Availability of FAQs
▪ Phone line/ virtual support community with knowledgeable moderator

## Discussion

This structured discussion of presbyopia from the perspective of people living with the condition provided a number of clear messages. Far from accepting it as a consequence of ageing, the participants called for an action-oriented view of presbyopia as a condition which may in future be effectively treated. The use of terms such as ‘normal’ or ‘natural’ was considered defeatist, shaping the attitude that affected individuals need to ‘*bear it and carry on*’. The experience of reduced quality of life from living with presbyopia drove a desire for an improved toolkit to deal with the condition. The substantial impact of presbyopia on participants’ quality of life in developed countries is in agreement with reports that although quality of life can be improved with visual corrective aids, it cannot currently be restored to the status before developing presbyopia [23].

The size of the panel, with nine participants with presbyopia and two HCP representatives, may appear modest. However, scoping out of the impact of presbyopia on individuals often starts with small samples: in the development of the new Presbyopia Impact and Coping Questionnaire (PICQ) the initial concept elicitation interviews were conducted with 20 participants [24]. Quantitative findings provide valuable guidance for further research and for the development of patient-relevant improvements to treatment. The common experiences and needs of the participants in the roundtable, who represented a number of countries and local conditions, speak for a similar impact of presbyopia across much of the globe.

Throughout history, presbyopia has been associated with ageing [11]. The need to break this connection in the minds of HCPs and the general public was voiced very strongly. In fact, it has long been known that decline in lens accommodation response begins as early as the first decade of life [11, 25]. Some members of the current panel developed presbyopia at 30–45 years; much younger ages than what is usually considered ‘elderly’. As with the question whether to consider the condition as a disease, (however defined) the views reflected a dissatisfaction with the sense of inevitability and normality attached to presbyopia.

The HCPs contacted by people with presbyopia were mainly optometrists, ophthalmologists and opticians. In their initial interactions with HCPs, there had been no discussion of alternative therapies to spectacles or contact lenses, perhaps reflecting the prevalent view that those affected by presbyopia should accept and adapt, with treatment focusing on non-medical solutions. This is in contrast to the quite active research interest in presbyopia. In addition to the miotics and lipoic acid choline esters discussed at the roundtable, there have been reports on improved lens elasticity and accommodation from anti-oxidant supplementation, resveratrol, lactic acid bacteria or periocular warming [26–28]. A greater awareness of emerging alternatives to current visual aids may empower those with presbyopia in their discussions with HCPs.

The innovative meeting format illustrated the value of interactive discussions between people with healthcare issues and specialist physicians. Patients and physicians alike have a large number of conflicting demands on their time, but as a virtual meeting, both groups were able to join the discussion at their own convenience. The format may find future use as a tool to counter the panel’s expressed lack of informed dialogue between patients and HCPs about conditions and therapeutic options.

The potential of new medical treatments was welcomed in the roundtable. Participants emphasised the need to reduce the inconvenience of their current visual aids. This need for greater convenience was also the reason for some scepticism towards the scenario of adding drops without removing the need for glasses. Easily administered drops and long-acting effects would increase convenience for subjects who did not need glasses for other conditions. But views on emerging medical treatments were coloured by pragmatism and a sense what several different options may have a place, depending on each individual’s need for corrective lenses and position on the presbyopia pathway.

Convenience was the guiding principle also in the panel’s consideration of clinical trial design. Beyond the need to schedule the number and times of clinic visits for minimal impact on participants’ schedules and a simplifications of testing regimens, a tool for peer-to-peer sharing of information was suggested to reduce discontinuation. This would presumably have to be restricted to practical matters of study conduct and safety, to avoid interfering with the outcomes of a study.

Healthcare authorities and clinical researchers are increasingly integrating patient-centred approaches and shared decision-making into their priorities [29]. The inclusion of adequate PROs and validated QoL questionnaires created for the purpose is indispensable for contemporary study analysis plans [16]. Most clinical trials of medical therapies for presbyopia have been of relatively modest size and focused on improved vision or accommodation. These are readily quantifiable measures and important outcomes of a therapy, but leave questions about their impact on subjects’ quality of life and other PROs unanswered.

Existing instruments to assess quality of life in presbyopia [30–33] tend to have shortcomings, e.g., inadequate documentation of content validity, poor psychometric measurement properties or a lack of focus. Whether the recently developed PICQ will live up to its promises of capturing those presbyopia-related impacts and coping behaviours which are important and relevant to patients [24] remains to be seen.

The format for the pooling of perspectives has limitations. In order to enable a structured roundtable discussion, the number of participants was limited and the sample may not adequately represent the experiences of all people living with presbyopia of different severity and in different environments. Likewise, the two participating HCPs presented their own views and interpretation of the medical situation, which may not always represent consensus views among HCPs globally. Although a number of questions were structured, the qualitative statements cannot be quantified and the findings will need to be confirmed in further studies, preferably using a number of different research methods. Finally, surgical alternatives were not a discussion topic.

In summary, this interactive exchange provided important insights into the how people with presbyopia cope with the condition, their focus on reducing the inconvenience presbyopia imposes on daily life, and the need to free presbyopia from the image of an inevitable companion of ageing which will need to be borne stoically by the affected person. The main topic of the roundtable was life experience and unmet needs, with HCPs taking an observatory role unless prompted. This innovative format would seem equally valid for an exploration of ophthalmologist and optometrist needs.

## Data Availability

The data that support the findings of this study are available from Novartis AG but restrictions apply to the availability of these data, which were used under license for the current study, and so are not publicly available. Data are however available from the corresponding author upon reasonable request and with permission of Novartis AG.
